# Task-Specific Effect of Transcranial Direct Current Stimulation on Motor Learning

**DOI:** 10.3389/fnhum.2013.00333

**Published:** 2013-07-01

**Authors:** Cinthia Maria Saucedo Marquez, Xue Zhang, Stephan Patrick Swinnen, Raf Meesen, Nicole Wenderoth

**Affiliations:** ^1^KU Leuven, Kinesiology and Rehabilitation Sciences, Research Center for Movement Control and Neuroplasticity, Heverlee, Belgium; ^2^PHL University College, Rehabilitation Research Institute, REVAL, UHasselt, Belgium; ^3^Neural Control of Movement Lab, Department of Health Sciences and Technology, ETH Zurich, Switzerland

**Keywords:** neuromodulation, tDCS, corticospinal excitability, primary motor cortex, motor learning, consolidation

## Abstract

Transcranial direct current stimulation (tDCS) is a relatively new non-invasive brain stimulation technique that modulates neural processes. When applied to the human primary motor cortex (M1), tDCS has beneficial effects on motor skill learning and consolidation in healthy controls and in patients. However, it remains unclear whether tDCS improves motor learning in a general manner or whether these effects depend on which motor task is acquired. Here we compare whether the effect of tDCS differs when the same individual acquires (1) a Sequential Finger Tapping Task (SEQTAP) and (2) a Visual Isometric Pinch Force Task (FORCE). Both tasks have been shown to be sensitive to tDCS applied over M1, however, the underlying processes mediating learning and memory formation might benefit differently from anodal transcranial direct current stimulation (anodal-tDCS). Thirty healthy subjects were randomly assigned to an anodal-tDCS group or sham-group. Using a double-blind, sham-controlled cross-over design, tDCS was applied over M1 while subjects acquired each of the motor tasks over three consecutive days, with the order being randomized across subjects. We found that anodal-tDCS affected each task differently: the SEQTAP task benefited from anodal-tDCS during learning, whereas the FORCE task showed improvements only at retention. These findings suggest that anodal-tDCS applied over M1 appears to have a task-dependent effect on learning and memory formation.

## Introduction

Repeated practice of a motor task induces skill learning, which is manifested as increasingly efficient movement execution (Luft and Buitrago, 2005). Depending on the task, skill learning might require days, weeks, or months of training. For example, a simple motor task that involves sequential finger movements will show improvements within minutes (Karni et al., 1995) while learning to play an instrument will require years of frequent practice (Dayan and Cohen, 2011). Once the motor task is acquired, performance can diminish again over time if practice is discontinued (Luft and Buitrago, 2005; Reis et al., 2009). At least three different but interconnected processes have been identified during motor skill learning: (1) practice effects resulting in performance gains during a training session (online gains); (2) memory consolidation occurring between sessions, i.e., during a period of rest or sleep making the acquired memory resistant against interference (offline effects) (Robertson et al., 2005; Reis et al., 2009), and (3) long-term memory formation as indicated by successful retention after days, weeks, or months (long-term retention).

Neural models of motor learning have generally converged toward the notion that different brain regions are recruited depending on the learning phase and the acquired task. Practice resulting in fast online gains triggers neuroplastic changes within the primary motor cortex (M1) and premotor areas, which are additionally modulated by executive functions and reward mechanisms. These changes are characterized by increased prefrontal activity, typically observed early in learning (Sanes, 2003), and by error-correction processes mediated by the cerebellum (Penhune and Steele, 2012). Interestingly, these mechanisms seem to be rather independent of the practiced motor task. By contrast, it has been proposed that consolidation of motor memories involves areas which are task-specific such that sequence learning relies strongly on cortico-striatal circuits, whereas sensorimotor adaptation relies predominantly on cortico-cerebellar pathways (Doyon and Benali, 2005; Doyon et al., 2009). If training continues over a longer period of time, movements become increasingly automatized and long-term representations are formed which are thought to rely on M1, premotor, and parietal areas (Doyon and Benali, 2005; Doyon et al., 2009; Penhune and Steele, 2012).

Recent studies have shown that anodal transcranial direct current stimulation (anodal-tDCS) results in increased corticomotor excitability when M1 is stimulated at rest (Nitsche and Paulus, 2000). Applying anodal-tDCS to M1 during online or offline processes has been shown to facilitate motor learning despite methodological differences across studies (for an overview, see Table 1). Interestingly, the beneficial effect of anodal-tDCS has been demonstrated for a large number of motor tasks (as shown in Table 1) such as explicit finger sequencing (Stagg et al., 2011), serial reaction time tasks (SRTTs) (Nitsche et al., 2003; Tecchio et al., 2010; Kantak et al., 2012), sequential visual isometric pinch force control (Reis et al., 2009; Schambra et al., 2011), the Jebsen Taylor hand function test (Boggio et al., 2006), ballistic thumb movements (Galea and Celnik, 2009), and reaching adaptation tasks (Galea et al., 2011). For serial reaction time and sequential pinch force control tasks it was further shown that applying anodal-tDCS to premotor areas has similar facilitating effects on learning (Kantak et al., 2012; Vollmann et al., 2013).

**Table 1:** **Summary of anodal-tDCS studies stimulating primary motor cortex aiming to improve motor sequence learning in healthy populations (all in right-handed participants)**.

Studies (year)	Task	Timing of Stim	Hand tested	*N* per group (years of age)	Montage of reference electrode	Electrode size	Current density intensity/duration	Training duration	Process facilitated by anodal-tDCS
Present study	SEQTAP (five digits)	Online	Left	13 (*24.6* ± 3.3)	Ipsilateral	*A* = 25 cm^2^; *R* = 99 cm^2^	0.04 mA/cm^2^; 1 mA/20 min	20 min of 40 s tap 20 s rest	Learning gains (online + offline) and long-term retention in a task-dependent manner.
Kantak et al. (2012)	Implicit SRTT (10 digits)	Online	Left	13	Contralateral	A = 8 cm^2^; *R* = 48 cm^2^	0.125 mA/cm^2^; 1 mA/15 min	?	Online and 1-day retention
Boggio et al. (2006)	JTT Hand; function test	Offline	Right and left	8 Females (22–26)	Contralateral	A = 35 cm^2^; R = 35 cm^2^	0.028 mA/cm^2^; 1 mA/20 min	10 Trials of JTT	Offline
Zimerman et al. (2013)	SEQTAP (five digits)	Online	Right	10 Old (68 ± 3.2); 10 young (25.2)	Contralateral	A = 25 cm^2^; R = ?	0.04 mA/cm^2^; 1 mA/20 min	Five blocks; 3 min train and 2 min rest	Online for the old group only
Stagg et al. (2011)	Explicit SRTT (10)	Online	Right	7 (22–31)	Contralateral	A = 35 cm^2^; R = 35 cm^2^	0.028 mA/cm^2^; 1 mA/15 min	15 min-3trials 12 s rest	Online
Tecchio et al. (2010)	SEQTAP (nine digits)	Offline	Left	22 (29 ± 5.3)	Ipsilateral	A = 35 cm^2^; R = 35 cm^2^	0.028 mA/cm^2^; 1 mA/15 min	10 min of 30 s per 10 s rest	Offline
Nitsche et al. (2003)	SRTT (12)	Online	Right	20 (23–34)	Contralateral	A = 35 cm^2^; R = 35 cm^2^	0.02 mA/cm^2^; 1 mA/15 min	15 min; 8 blocks; 120 trials	Online
Reis et al. (2009)	FORCE	Online	Right	12 (28.3 ± 2.2)	Contralateral	A = 25 cm^2^; R = 25 cm^2^	0.04 mA/cm^2^; 1 mA/20 min	45 min; 200 trials	Offline and long-term retention
Galea and Celnik (2009)	Thumb movements	Online	Right	9 (30 ± 9)	Contralateral	A = 25 cm^2^; R = 25 cm^2^	0.04 mA/cm^2^; 1 mA/30 min	1 Hz for 30 min	Offline
Galea et al. (2011)	Visuomotor adaptation	Online	Right	10 (27 ± 6)	Contralateral	A = 25 cm^2^; R = 25 cm^2^	0.08 mA/cm^2^; 2 mA/15 min	25 × 200 trials	Retention but did not affect online
Vines et al. (2008)	SEQTAP (five digits)	Offline	Right and left	17	Contralateral	A = 16.3 cm^2^; R = 30 cm^2^	0.06 mA/cm^2^; 1 mA/20 min	3 × 30 s × 30 s break	Different offline effect depending on the target hemisphere (right/left)
Vines et al. (2006)	SEQTAP (five digits)	Offline	Right and left	7	Contralateral	A = 15 cm^2^; R = 30 cm^2^	0.06 mA/cm^2^; 1 mA/20 min	3 × 30 s × 30 s break	Different offline effect depending on the hand (right/left) tested
Schambra et al. (2011)	FORCE	Online	Right and left	14 (27.8 ± 0.6)	Ipsilateral	A = 25 cm^2^; R = 25 cm^2^	0.04 mA/cm^2^; 1 mA/20 min	Six blocks 200 trials	Learning gains (online + offline).

*Task abbreviations: SRTT, serial reaction time task; SEQTAP, serial sequential finger tapping task; JTT, Jebsen Taylor hand function test; FORCE, sequential visual isometric pinch force task. Montage of the reference electrode: contralateral = reference electrode placed over the contralateral supraorbital area; ipsilateral = reference electrode placed over the ipsilateral arm. Electrode size abbreviations: A, anodal; R, reference electrode*.

Two of these previously mentioned transcranial direct current stimulation (tDCS) studies have compared all three learning processes directly (Reis et al., 2009; Kantak et al., 2012) but revealed divergent results even though both studies applied tDCS during the training sessions (i.e., targeting online gains): Reis et al. (2009) showed that anodal-tDCS enhanced skill acquisition of an *isometric pinch force task*, which involved learning to control a force transducer in order to move a cursor displayed on a computer screen through a sequence of five horizontal targets. Training of this task occurred over a period of 5 days. The beneficial effect of anodal-tDCS mainly emerged through an effect on offline gains between sessions (offline effects), and was still present at a long-term retention test (RT) taking place up to 3 months after acquisition. Conversely, a *SRTT*, in which a sequence of key-presses was cued visually, did not show offline effects (Kantak et al., 2012). Instead, anodal-tDCS boosted behavioral improvement during training (online gains) which resulted in improved performance on a RT 1 day later.

This previous work suggests that applying anodal-tDCS to M1 influences online, offline, and long-term retention processes in a task-specific manner. This is in line with the proposal that depending on the task characteristics and attentional demands (Hazeltine et al., 1997), online and offline processes occurs either predominantly in M1 or rely critically on neuroplasticity in other areas such as the cerebellum or basal ganglia. Evidence for task-specificity of tDCS was revealed by Galea et al. (2011) who showed that a visual adaptation task was differentially influenced by anodal-tDCS over M1 versus the cerebellum. Alternatively, it could be argued that the differences between the studies of Reis et al. (2009) and Kantak et al. (2012) were due to a difference in training duration that consisted either of multiple days of practice (Reis et al., 2009) or one single training session (Kantak et al., 2012). A lack of multisession tDCS studies in the field of motor skill learning, and methodological differences regarding stimulation parameters makes it difficult to draw clear conclusions on these controversial results.

Here, we investigate whether the beneficial effect of anodal-tDCS on online gains, offline effects, and long-term retention after multiple days of motor practice is task-specific. Each participant acquired a modified version of the sequential isometric visual pinch force task (FORCE) similar to that of Reis et al. (2009) and a Sequential finger tapping task (SEQTAP) (Walker et al., 2003). The tasks were trained either with anodal-tDCS applied over M1 (experimental group) or with sham-tDCS (control group). Both tasks have been previously presented in the tDCS literature but, to the best of our knowledge have never been directly compared to each other over multiple sessions. In particular, we tested the effects of tDCS on the three different phases influencing motor skill learning (i.e., online gains, offline effects, and long-term retention).

Based on previous research (Nitsche et al., 2003; Reis et al., 2009; Schambra et al., 2011; Stagg et al., 2011; Kantak et al., 2012) we predicted that the anodal-tDCS group would show enhanced motor skill learning compared to the control group in both tasks. However, it is uncertain whether anodal-tDCS effects on (i) online gains, (ii) offline gains, or (iii) long-term retention are task-specific because motor skill learning represents a complex cognitive process relying on a number of different areas in addition to M1, such as premotor and supplementary motor cortex, the cerebellum and the basal ganglia (Ungerleider et al., 2002). Further understanding of how tDCS affects motor skill learning could have clinical implications, as tDCS is increasingly considered as an adjuvant therapy for patients with motor deficits, for example after stroke (Hummel et al., 2005; Adeyemo et al., 2012; Madhavan and Shah, 2012).

## Materials and Methods

### Ethics statement

Study protocol and informed consent were approved by the local Ethics Committee for Biomedical Research at the Katholieke Universiteit Leuven, in agreement with the Code of Ethics of the World Medical Association (Declaration of Helsinki) (Rickham, 1964). Written informed consents were obtained from all subjects prior to participation. Financial compensation was given for participation in this study.

### Subjects

Thirty healthy subjects (15 females, age 23.9 ± 3.13 years) participated in this study. All participants were right-handed, as assessed by the Oldfield Questionnaire (Oldfield, 1971) (scores 87.73 ± 18.73%). They were naive to the purpose of the study and none had sensorimotor or neurological deficits (self reported). Each volunteer was screened for risk factors and potential adverse effects caused by transcranial magnetic stimulation (TMS)/tDCS as well as for skin abnormalities at the proposed electrode sites (cut, abrasion, rash) and excluded when necessary. Two subjects dropped out before completion of the study, and one was excluded because of learning gains exceeding more than two standard deviations above the group mean. The remaining 27 subjects (14 in the experimental group and 13 in the control group; 6 females in each group) were included in the statistical analysis. At the beginning of the experiment subjects reported their level of physical activity for the period 2 h prior to the experiment: low, moderate, or high (American College of Sports Medicine, 2006). There were no significant differences in activity levels between groups (*p* = 0.82). An overview of baseline characteristics is given in Table 2.

**Table 2:** **Psychometric data**.

	SEQTAP			FORCE		
	Mean anodal (14)	Mean sham (13)	*p*-Value	Mean anodal (14)	Mean sham (13)	*p*-Value
Age (years)	23.14 ± 2.6	24.85 ± 3.51	0.16	23.14 ± 2.60	24.85 ± 3.51	0.16
Handedness (%)	92.3 ± 12.35	83.15 ± 23.1	0.22	92.3 ± 12.35	83.15 ± 23.1	0.22
Sleep day 1 (hours)	6.57 ± 1.22	7.29 ± 0.89	0.1	6.97 ± 1.68	6.57 ± 0.79	0.46
Sleep day 2 (hours)	7.43 ± 1.19	6.58 ± 1.08	0.07	7.07 ± 0.73	6.65 ± 1.91	0.46
Sleep day 3 (hours)	6.93 ± 1.30	6.83 ± 0.94	0.83	7.12 ± 1.33	6.80 ± 1.09	0.52
Sleep day 4 (hours)	7.29 ± 0.99	6.83 ± 1.07	0.28	7.15 ± 0.83	7.04 ± 0.63	0.69
Sleep quality^†^ (0–10)	7.68 ± 0.95	6.95 ± 1.71	0.19	7.69 ± 0.95	6.65 ± 2.07	0.11
Attention^†^ (0–10)	8.39 ± 1.11	8.42 ± 1.08	0.96	8.38 ± 0.94	7.69 ± 1.39	0.15
Fatigue^†^ (0–10)	3.25 ± 1.87	4.21 ± 2.39	0.26	3.15 ± 2.41	2.04 ± 1.94	0.21
Discomfort^†^ (0–10)	1.61 ± 1.92	0.66 ± 1.72	0.2	2.27 ± 2.31	0.54 ± 0.69	0.01*

*^†^Scale from 0 to 10, where 0 is worst sleep quality/attention, no fatigue/discomfort, and 10 is best sleep quality/attention, maximal fatigue/discomfort*.

* p < 0.05.

### Overall study design

The present study investigated the effect of applying tDCS, either anodal or sham, to M1 during the acquisition of two different motor tasks: (1) SEQTAP and (2) FORCE. tDCS was applied in a double-blind, sham-controlled design.

**Figure 1:**
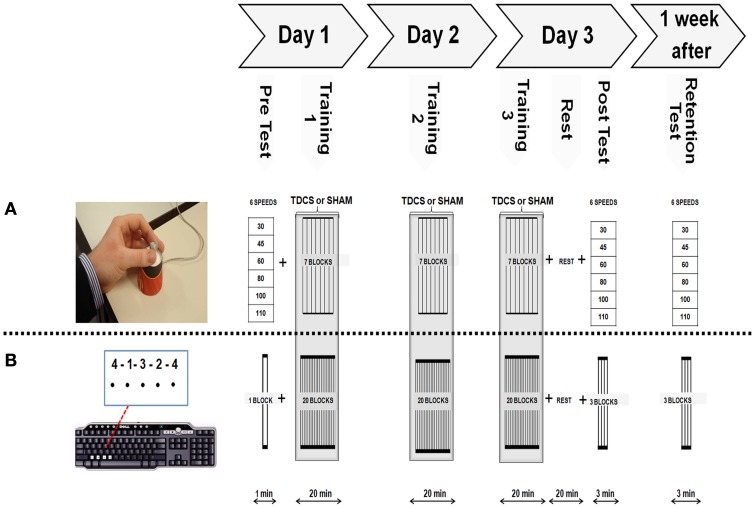
**Experimental Protocol**. Both motor tasks were acquired following the same systematic order as shown on top of this figure’s diagram. **(A)** Modified isometric pinch force task (FORCE) required subjects to control a force sensor with their left thumb and index finger to move a cursor to a series of target zones, as fast and accurately as possible. **(B)** The sequential finger tapping task (SEQTAP) required subjects to repeatedly tap a fixed five element sequence with their left hand, as fast and accurately as possible on a keyboard.

Subjects were randomly assigned to an anodal-tDCS or sham-tDCS group. Each group performed two different learning sessions separated by at least 2 months with the order of acquiring the SEQTAP and FORCE task counterbalanced across participants. Stimulation type (anodal versus sham) was kept consistent for each subject in both sessions. Each experimental session consisted of a pre-test (PRE) at day 1, 20 min of motor practice for three consecutive days while either anodal-tDCS or sham-tDCS was applied (Day 1, Day 2, and Day 3), a post-test (POST) 20 min after completion of training on Day 3 and a RT 1 week after the last training session (Figure 1). Instructions, feedback, and motivation were standardized for all sessions. At the beginning of each training session subjects were familiarized with the motor task. Questionnaires using visual analog scales (VAS) (Floel et al., 2004) were performed to evaluate subject’s perception of attention and fatigue before and after anodal-tDCS and sham-tDCS sessions. At the end of each session participants were asked to describe their sense of discomfort/pain. Each session lasted between 60 and 90 min.

### Sequential finger tapping task

Participants were seated on an office-chair approximately fifty centimeters away from a computer screen, with the non-dominant arm performing the task in a supported position. A five-element sequence (4-1-3-2-4) was displayed on the computer screen. Each number indicated a key press with the index (1), middle (2), ring (3), or little finger (4), respectively, which was registered by a computer keyboard (Figure 1B lower panel). Subjects did not receive any feedback regarding performance and the only visual information presented was a black dot appearing underneath each number to indicate that the key press had been recorded (Figure 1B lower panel). Immediately after pressing the last key to complete the five-digit sequence, all dots were removed indicating the start of a new practice sequence. Thus, the visual sequence was permanently displayed on the screen during the entire 40 s practice trial and subjects were instructed to complete as many sequences as they could without a pause. The task was presented with E-prime software (Psychology Software Tools, Inc.). Subjects had to tap the sequence as quickly and accurately as possible for a period of 40 s which was followed by 20 s rest (i.e., task/rest ratio = 2:1). The PRE measurement at baseline consisted of one trial (1 min total). This short duration was chosen because this task is characterized by large initial improvements which might bias PRE performance when included. For the training sessions, 20 trials (20 min) were performed each day. The POST and RT measurements consisted of three trials each (3 min total). All subjects practiced the same sequence throughout all experimental sessions.

### Isometric force control task (FORCE)

The FORCE task was slightly modified from a previous version developed by Reis et al. (2009). Subjects were required to control a cursor displayed on a computer monitor with a force transducer using the thumb and index finger of their non-dominant hand (Figure 1A upper panel). They had to move the cursor (green square) between the home position (force = 0) and nine target zones that were visited in a fixed order (6 3 1 7 2 9 5 8 4) (Figure 1A upper panel). The targets were equally distributed over the screen corresponding to 100 virtual units (VU). The size of the cursor corresponded to 5 VU and the width of each target zone (indicated by two vertical lines) corresponded to 10 VU. The amount of pinch force applied to the sensor was non-linearly related to the displacement of the cursor according to the formula: screen Position = *a* × ln (force) + *b* with *a* and *b* adjusted such that reaching the furthest target required ∼40% of the Maximal Voluntary Contraction (MVC). MVC was taken as the highest value of three maximal pinch force trials at the beginning of the experiment.

The protocol for acquiring the FORCE task was constructed in the same way as the protocol of the SEQTAP task: a PRE measurement at baseline, three consecutive days of training followed by a POST-test (20 min after training at day 3), and a RT after 1 week. For the PRE, POST, and RT measurements, subjects performed the FORCE task while being paced at six different speeds (30, 45, 60, 80, 100, 110 bpm), with a metronome. Each speed was repeated twice in a random order during the pre measurement, and three times for the POST and RT measurements. Subjects were instructed to perform the task as accurately as possible. Again the PRE measurement was shorter than POST and RT measurements to limit the influence of large improvements typically seen at the beginning of learning.

The training session was divided into seven blocks, each block consisting of 2 min performing the FORCE task and 1 min of rest (i.e., task/rest ratio = 2:1), with a total duration of 20 min per session. Training was performed over three consecutive days. Execution speed was chosen by the subject, who was always instructed to perform the task as fast and accurate as possible.

### Transcranial magnetic stimulation

Transcranial magnetic stimulation was used to localize the hand representation in M1. TMS was delivered via a figure-of-eight coil (70 mm diameter) connected to a Magstim 200 Stimulator (Magstim, Whitland, Dyfed, UK). The coil was positioned over the right hemisphere such that the handle pointed backwards and away from the midline at an angle of 45°. This position ensured a posterior-lateral to anterior-medial flow of the induced current approximately perpendicular to the central sulcus, which is optimal for stimulating the corticospinal pathway of M1. Motor evoked potentials (MEPs) were recorded by an electromyogram (EMG) measured by two disposable Ag-AgCL surface electrodes (Blue sensor SP) placed over the first dorsal interossei (FDI) muscle in a belly-tendon montage and a reference electrode placed over a bone. TMS was used to determine the so-called “hotspot,” i.e., the position where MEPs with the highest and most consistent amplitudes were evoked in the left FDI muscle. TMS triggering and EMG recordings were controlled by Signal Software (4.0 Version, Cambridge Electronic Design, UK).

### Transcranial direct current stimulation

Transcranial direct current stimulation was applied via two silicone electrodes connected to a battery-driven stimulator (HDCStim class IIa; Model:HDCel EN-05, Newronika s.r.l., Milano 20122, Italy). The active “anode” (5 cm × 5 cm) was covered with an equally distributed amount of conductive gel and located over the FDI hotspot of right M1. The right hemisphere was selected because of the greater possibility of observing further improvements with the less often used non-dominant hand. Since we used a 3-day training protocol performance might plateau too early when the dominant hand would have been used, as observed in the study by Boggio et al. (2006). These authors found a positive effect of anodal-tDCS only when applied to the non-dominant motor area but not to the dominant motor area due to early ceiling effects. The cathodal reference electrode was covered by a sponge (11 cm × 9 cm) soaked in saline solution and positioned on the ipsilateral shoulder. Using a bigger electrode size makes the reference electrode functionally inefficient, due to a spreading of the current, as previously demonstrated by Nitsche et al. (2007). The placement of the cathodal electrode was chosen to prevent additional effects on other areas (e.g., prefrontal cortex) which might be also involved in motor memory formation. Good conductivity of both electrodes was ensured to minimize the risk of burns (Palm et al., 2008) or other skin irritations (Riedel et al., 2012). Placement of each electrode was marked with a waterproof pen to guarantee identical positions during the whole course of the experiment. For anodal-tDCS stimulation a constant current of 1 mA was delivered for 20 min. For sham-tDCS stimulation the same current was applied but only for the first 30 s. All subjects and investigators were blind regarding the tDCS intervention.

### Data analysis

Most motor tasks are affected by the so-called speed-accuracy tradeoff, i.e., accuracy decreases when speed increases and vice versa. A skill index (SI) considers both parameters such that it increases, for example, when movements are performed with the same accuracy but with higher speed or with higher accuracy but the same speed. For the SEQTAP task previous work from our lab indicated that there is a linear relationship between accuracy and speed. Thus the SI for the SEQTAP task was calculated by:

$${\text{SI}}_{\text{SEQTAP}}=\frac{\text{\% Correct}\;\text{Sequences}}{\text{mean}\;\text{response}\;\text{time}\;\text{per}\;\text{each}\;\text{40s}\;\text{trial}}$$

In agreement with Reis et al. (2009), we determined the speed-accuracy tradeoff model of the FORCE task empirically. This was achieved by pacing each subject at different movement frequencies in order to model the associated changes in accuracy. For the FORCE task there was a non-linear relationship between accuracy and speed that is best modeled by:

$${\text{SI}}_{\text{FORCE}}=\frac{\text{1 - error rate}}{\text{error rate}\left(\text{ 1n}{\left(\mathrm{duration}\right)}^{b}\right)}$$

**Figure 2:**
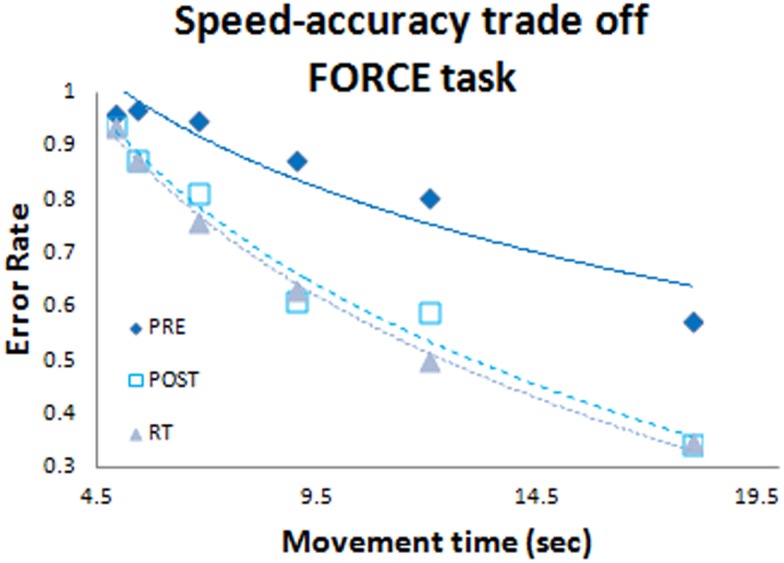
**Speed-accuracy tradeoff function**. Blue diamonds represent the pre test data set; white squares represent the post test data set; and grey triangles represent the retention data set. Data were included from all subjects (both anodal-tDCS and sham-tDCS groups).

In previous work (Reis et al., 2009; Schambra et al., 2011) the *b*-value was set to *b* = 5.424, a constant value derived from a small group of subjects performing a control experiment (Reis et al., 2009). Here we determined accuracy of the FORCE task for different speeds at PRE, POST, and RT (Figure 2). The POST and RT data were used to determine the “*b*” value for each subject and this value was used to calculate the SI_FORCE_ across all days. On average our value (*b* = 5.14 ± 1.2 SD) was very similar to the value reported previously (*b* = 5.424, Reis et al., 2009). However, we observed large inter-individual difference across subjects with values ranging from 2.9 to 8.1. Thus, using individually determined *b*-values allows a more accurate reflection of FORCE task performance.

SI_FORCE_ and SI_SEQTAP_ values for each subject were averaged within PRE, POST, and RT sessions, respectively. Changes in SI due to learning gains (online and offline combined) were calculated by subtracting PRE from POST, and long-term retention gains were calculated by subtracting POST from RT. We also calculated the ratio of long-term retention gains relative to the combined learning gains [(RT-POST)/(POST-PRE)].

SI_FORCE_ and SI_SEQTAP_ during training were averaged within blocks. For the FORCE task training there were seven blocks a day. For the SEQTAP task there were 20 trials a day. To compare the training data across tasks, the SEQTAP task data was averaged across three trials (1–3, 4–6, 7–9, and so on) except for the second-last block (averaged over 16–17) to match the seven data points of the FORCE task. Also, since the units of the data differed between tasks, a *z*-transformation was applied.

From the training data we determined the online effects of the FORCE task by subtracting the first trial from the last trial on each day. Offline effects were quantified by subtracting the last trial on 1 day from the first trial of the subsequent day. The same procedure was used for the SEQTAP task, only that the average of the three first and three last trials was used.

### Statistics

For analyzing the learning gains (POST-PRE), and the retention gains (RT-POST), non-parametric statistics were used because SI_FORCE_ values deviated from normality, as indicated by Kolmogorov–Smirnov test (*p* > 0.002). Mann–Whitney *U*-tests were used to identify the stimulation effect by comparing performance gains between the anodal-tDCS and the sham-tDCS group. The within-subjects effect of TASK was analyzed by the non-parametric Friedman’s test. Additionally, Spearman correlation coefficients were calculated to compare the gains between tasks.

The training data were normally distributed and an Analysis of Variance for repeated measurements (repeated measures ANOVA) was used with the factors TIME (1–21 SI scores throughout 3-day practice), STIM (anodal-tDCS versus sham-tDCS), and TASK (SEQTAP versus FORCE). Also online and offline gains were normally distributed and subjected to a repeated measures ANOVA with the factors DAY, STIM, and TASK. Paired *t*-tests were used to evaluate the VAS scores of fatigue, attention, and discomfort/pain. The alpha-level was set to alpha = 0.05.

## Results

### Psychometric data

Psychometric measurements are summarized in Table 2 for each group and task. Most values are similar across groups and task and the only significant difference between the anodal-tDCS and the sham-tDCS group was found in rating discomfort for the FORCE task: the anodal-tDCS group reported higher values (more discomfort) than the sham-tDCS group. However, overall values were low and this result was not reproduced for the SEQTAP task. Further, subjects were instructed that subjective sensation can vary substantially between subjects and sessions. Therefore, it is expected that subjects of each group were sufficiently blinded regarding the intervention.

#### Does anodal-tDCS influence SEQTAP learning when comparing PRE, POST, and RT?

**Figure 3:**
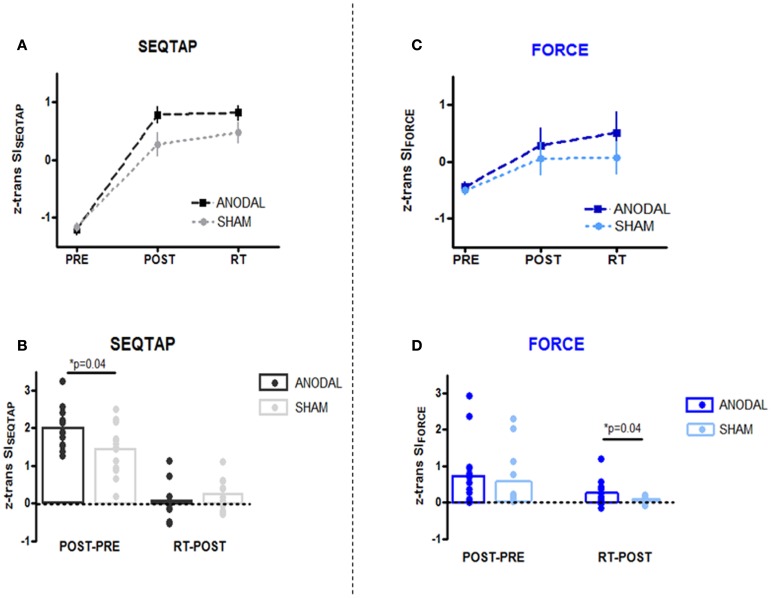
**PRE, POST, and RT measurements (upper panel) and learning/retention gains (lower panel)**. *Z*-transformed data from Skill Index (*z* trans SI) are shown for both tasks. **(A,B)** SEQTAP data: anodal-tDCS (black squares/bars) compared with the sham-tDCS (gray circles/bars). **(C,D)** FORCE data: anodal-tDCS (blue squares/bars) compared with the sham-tDCS (turquoise circles/bars).

Both the anodal-tDCS and sham-tDCS groups improved SEQTAP performance substantially from PRE to POST, while SI_SEQTAP_ remained nearly unchanged from POST to RT (Figure 3A). SI_SEQTAP_ learning gains (POST-PRE) were significantly higher for anodal-tDCS than sham-tDCS (*U* = 50, *Z* = 1.96, *p* = 0.04) while no significant stimulation effects were found for long-term retention (RT-POST) (*U* = 73.5, *Z* = −0.82, *p* = 0.41) (Figure 3B).

#### Does anodal-tDCS influence FORCE learning when comparing PRE, POST, and RT?

For the FORCE task, both groups improved from PRE to POST, but additional improvements were observed form POST to RT for the anodal-tDCS group (Figure 3C). Statistics revealed that while SI_FORCE_ gains did not differ significantly during learning (POST-PRE) (*U* = 77, *Z* = 0.65, *p* = 0.52), the anodal-tDCS group had significantly larger long-term retention gains (POST-PRE) (*U* = 77, *Z* = 0.65, *p* = 0.04) (Figure 3D).

#### Is the anodal-tDCS effect task-specific?

First we pooled the data across the anodal-tDCS and sham-tDCS groups and tested whether learning or long-term retention gains were correlated between tasks. The Spearman correlation analysis revealed only insignificant effects for both learning (*r* = 0.22, *p* = 0.26) and long-term retention gains (*r* = −0.038, *p* = 0.85). This indicates that task learning was largely independent.

As can be seen in Figure 3, learning gains were substantially smaller for the FORCE than the SEQTAP task even after SI values were *z*-transformed within each task. Therefore we compared the gain-ratios [(RT-POST)/([POST-PRE)] which were smaller for the SEQTAP task than for the FORCE task, but only in the anodal-tDCS group (SEQTAP = 0.05 ± 0.08 FORCE = 0.95 ± 0.46) and not in the sham-tDCS group (SEQTAP = 0.56 ± 0.45 FORCE = 0.073 ± 0.13). Note that small values indicate that gains became mainly expressed during the 3 days of practice rather than due to long-term retention. Friedman’s ANOVA revealed a significant main effect of TASK for the anodal-tDCS group [χ^2^(1) = 7.14, *p* = 0.007] but not for the sham-tDCS group [χ^2^(1) = 0.28, *p* = 0.59].

#### Does anodal-tDCS influence training of each task?

**Figure 4:**
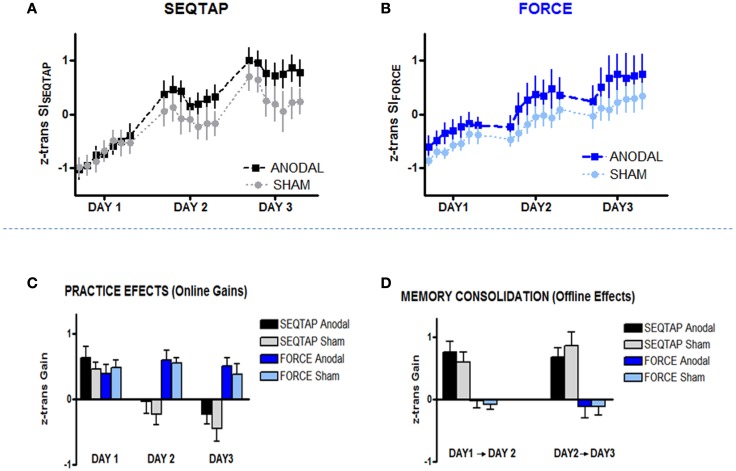
**Training Data**. *Z*-transformed data from Skill Index (*z* trans SI) are shown for both tasks. **(A)** SEQTAP Training overview for Day 1, Day 2, and Day 3 for anodal-tDCS (black squares) and sham-tDCS (gray circles) **(B)** FORCE training overview for each day between anodal-tDCS (blue squares) and sham-tDCS (turquoise circles) **(C)** Online gains for SEQTAP and FORCE data: anodal-tDCS (black and blue bars respectively) compared with the sham-tDCS (gray and turquoise bars respectively). We determined online effects for the FORCE task by subtracting the first trial from the last trial for each day. For the SEQTAP we used the same procedure, with the only difference that the average of the three first and the three last trials was used. **(D)** Offline effects in the SEQTAP and FORCE data: anodal-tDCS (black and blue bars respectively) compared with the sham-tDCS (gray and turquoise bars respectively). Offline gains in the FORCE task were quantified by subtracting the last trial of 1 day from the first trial of the subsequent day. The same procedure was used for the SEQTAP task, with the only difference that the average of the three first and the last three trials was used. Vertical bars indicate standard errors.

Over the 3 days of practice all subjects improved performance for both SEQTAP and FORCE tasks irrespective of which stimulation was applied (Figures 4A,B). This was further confirmed by a significant main effect of TIME (*F*_20, 500_ = 61.3, *p* < 0.001). However, over the course of training the anodal-tDCS group improved more strongly than the sham-tDCS group, indicated by a significant TIME × STIM interaction (*F*_20, 500_ = 1.85, *p* = 0.014). The overall gain was larger for the SEQTAP than for the FORCE task, indicated by a significant TASK × TIME interaction (*F*_20, 500_ = 7.24, *p* < 0.001). No other main effects or interactions reached significance (*p* > 0.11).

Further analysis revealed that the significant TIME × STIM interaction was mainly driven by the SEQTAP task, which exhibited a significant TIME × STIM interaction when the same repeated measures ANOVA was applied to the SEQTAP data only (*F*_20, 500_ = 1.88, *p* = 0.012; Figure 4A). By contrast, performing the same analysis for the FORCE data did not reveal a trend toward a significant TIME × STIM interaction (*F*_20, 500_ = 0.55, *p* = 0.944) as shown in Figure 4B.

#### Did anodal-tDCS influence practice (online effects) versus consolidation (offline effects) differently?

The two tasks differed significantly regarding gains observed due to practice (online effects) and memory consolidation (offline effects): for the SEQTAP task, online gains were only observed for day 1, while the SI_SEQTAP_ scores attenuated during practice at Day 2 and Day 3 (Figure 4C, black/gray bars). Offline-improvements from Day 1 to Day 2, as well as from Day 2 to Day 3 were substantial, as typically expected for this task (Figure 4D, black/gray bars).

By contrast, practicing the FORCE task resulted in robust online improvements for each of the 3 days (Figure 4C, blue/turquoise bars), while offline effects resulted either in unchanged or slightly diminished performance (Figure 4D, blue/turquoise bars).

Accordingly, statistics revealed a significant TASK and TASK × TIME interaction (*F*_20, 500_ ≥ 16.79, *p* < 0.001) for the online effects (Figure 4C), and a significant TASK effect (*F*_20, 500_ ≥ 68.77, *p* < 0.001) for the offline effects (Figure 4D). However, there were no significant main effects or interactions including the factor STIM.

## Discussion

We investigated whether anodal-tDCS effects are task-specific in relation to three different stages of motor skill learning: (i) online effects due to practice, (ii) offline effects due to consolidation, and (iii) long-term retention. Anodal-tDCS over M1 enhanced learning gains (comprising both online and offline effects) for the SEQTAP task, whereas long-term retention was improved for the FORCE task. These findings confirm our hypothesis that positive effects of anodal-tDCS were present during different learning phases depending on which motor task were acquired.

Our results confirm previous findings that anodal-tDCS is beneficial for motor learning (Nitsche et al., 2003; Boggio et al., 2006; Galea and Celnik, 2009; Reis et al., 2009; Tecchio et al., 2010; Galea et al., 2011; Schambra et al., 2011; Stagg et al., 2011; Kantak et al., 2012; Zimerman et al., 2013). Our most novel result is that the effect of anodal-tDCS is task-specific, with each motor task benefiting from anodal-tDCS at different learning stages. This may suggest that the contribution of M1 to different learning and memory related processes depends on the task acquired.

### Why does anodal-tDCS over M1 influence the two tasks differently?

In the present study we showed that anodal-tDCS over M1 differentially affected learning of the SEQTAP and FORCE tasks: anodal-tDCS facilitated learning gains in the SEQTAP task and long-term retention in the FORCE task. Accordingly, the ratio of long-term retention gains (RT-POST) versus learning gains (POST-PRE) was significantly larger for the FORCE than for the SEQTAP task, but only in the anodal-tDCS group. Consistent with our interpretation, anodal-tDCS facilitated significant performance gains during training only in the SEQTAP task. While the FORCE task also benefited from anodal-tDCS during training, this effect did not reach significance and was not robust across subjects.

For the SEQTAP task it has been argued that distinct brain areas serve different learning stages: according to Doyon et al. (2009), early learning involves predominantly M1 while early consolidation relies strongly on the striatum that supposedly mediates the formation of chunks, an important feature for speeding up tapping performance (Doyon et al., 2009). Based on this model it is not surprising that anodal-tDCS over M1 had little effect on consolidation since the striatum is located underneath the cortex, too deep in the brain to be reached by tDCS. Also, the FORCE task has a sequential element (different targets need to be visited in a fixed order). However, accurate force control is probably the most important sensorimotor parameter determining success. It is well known that M1 neurons play an important role in force control (Ashe, 1997; Keisker et al., 2009; Sulzer et al., 2011). Accordingly, one can hypothesize that not only learning, but also consolidation and long-term retention of improved force control rely predominantly on M1 neurons. Therefore, it is likely that anodal-tDCS over M1 influences all learning mechanisms including consolidation and long-term retention for the FORCE task.

In summary, our findings suggest that anodal-tDCS had a stronger influence on neurons located in M1, i.e., directly underneath the electrodes rather than on more remote areas interconnected with M1, such as the striatum. Therefore, the facilitating effect of anodal-tDCS on motor learning appears to depend on the degree to which M1 is involved in memory formation (Sanes, 2000; Sanes and Donoghue, 2000).

### Anodal-tDCS effect on SEQTAP task

For the SEQTAP data, anodal-tDCS facilitated memory formation over the course of training. This was indicated by both significantly larger performance gains from PRE to POST and larger improvements during the training sessions from Day 1 to Day 3 for the anodal-tDCS group. However, significance was only reached when online and offline effects were combined since neither measurement reached significance when tested in isolation. This is generally in line with previous work demonstrating beneficial effects of anodal-tDCS on similar sequential tapping tasks. Decreased RT’s have previously been observed in subjects receiving anodal-tDCS (Nitsche et al., 2003; Stagg et al., 2011; Kantak et al., 2012). However, in these studies the effect of anodal-tDCS had already emerged during the first practice session, while we observed benefits of the stimulation only after 3 days of training. There are several explanations for these divergent results.

First, the aforementioned studies tested a SRTT, which requires subjects to respond as quickly as possible to a visual cue. An important component of learning in this task is gaining an implicit knowledge of the cued sequence. By contrast, the SEQTAP task requires preprograming and executing a sequence of finger taps as quickly as possible, thereby not relying on external stimuli. Using a paradigm similar to ours, Zimerman et al. (2013) did not find an effect of anodal-tDCS when applied during a 1-day training session in young subjects.

Second, all of these studies placed the cathodal electrode over the contralateral supraorbital area (Nitsche et al., 2003; Stagg et al., 2011; Kantak et al., 2012; Zimerman et al., 2013) while we placed it over the ipsilateral shoulder (as shown in Table 1 and discussed below in further detail). This montage might have resulted in a smaller tDCS effect (Schambra et al., 2011).

Finally, our training protocol required subjects to tap the sequence during continuous 40 s blocks. This might have influenced our SI measure since Brawn et al. (2010) have shown that this form of massed training can cause a phenomenon called reactive inhibition, defined as a decrease in performance due to extensive training. This effect can be observed within trials lasting longer than 10 s. As shown in Figure 4, SI_SEQTAP_ decreased during training on Day 2 and Day 3 which was followed by substantial offline gains when subjects returned the next day. This pattern indicates that reactive inhibition might have masked true skill performance at the end of training. It is possible that the benefit of anodal-tDCS is derived from a release of reactive inhibition. Note, however, that the POST session was performed 20 min after practice such that effect of reactive inhibition was minimal. Importantly, there were significantly larger gains from PRE to POST for the anodal-tDCS group than for the sham-tDCS group, indicating a real improvement in skill performance.

### Anodal-tDCS effect on FORCE task

For the FORCE task we found a positive effect of anodal-tDCS on long-term retention only, suggesting that anodal-tDCS had a more robust influence on offline memory formation than on immediate training processes. These findings are partly in line with previous studies using the same task and reporting an overall beneficial effect of anodal-tDCS (Reis et al., 2009; Schambra et al., 2011). However, Reis et al. (2009) reported that anodal-tDCS was most beneficial for offline effects, a result we did not reproduce. In addition to minor differences in the task (we used nine targets as opposed to five), the electrode montage used here was less efficient than that applied by Reis et al. (2009). This argument is further supported by Schambra et al. (2011) who used the same montage as we did and also report a much smaller effect size than (Reis et al., 2009) Reis and Fritsch (2011) (see below for a detailed discussion).

In summary, previous work and our results suggest that anodal-tDCS applied during the FORCE task is mostly beneficial to offline consolidation processes, either over night (Reis et al., 2009) or when testing long-term retention. This proposal is further supported by other work stressing the importance of M1 for motor memory consolidation (Muellbacher et al., 2000; Galea and Celnik, 2009).

### Limitations of the study

Our results are generally in line with previous reports on the beneficial effect of anodal-tDCS over M1 on motor learning, underlining that this technique has great potential, for example, in neurorehabilitation.

However, pinpointing which mechanism might underlie the beneficial effect of anodal-tDCS is difficult because consistency across studies is relatively low. This might be a result of the small sample sizes used here and elsewhere (median *n* = 12 per group, see Table 1 for an overview) and/or from large inter-individual differences. That is, some volunteers respond strongly to tDCS while others show no effect. First, it is important to note that only some of the applied tDCS current passes through the brain, with as much as half of it being shunted through scalp tissue (Miranda et al., 2006; Sadleir et al., 2010). It has also been suggested that tDCS current might differentially affect neurons based on their orientation and morphology (Radman et al., 2009). Thus, individual anatomy such as the amount and conductivity of cerebrospinal fluid, the percentage of fat tissue underneath the skin, thickness of the skull, head size, and the orientation of the neurons and gyri at the site underneath the electrode are likely to impact strongly on the response to non-invasive brain stimulation techniques, including anodal-tDCS (Conde et al., 2012; Datta et al., 2012; Truong et al., 2012). This would imply that stimulating all individuals with an intensity of 1 mA is not ideal, because depending on the sample, a large number of non-responders may have contributed to inconsistencies across studies. Therefore, an individualized tDCS protocol utilizing neurophysiological data or computer models would be optimal to test the effects of tDCS and to guide the optimization of clinical trials and electrotherapy.

Another challenge is that stimulation parameters (i.e., intensity and duration) and electrode montage differed substantially across studies (see Table 1) making it difficult to draw general conclusions. Regarding stimulation intensity, Parazzini et al. (2011) showed that the efficacy of tDCS depends on current density since it determines the induced electrical field strength (Parazzini et al., 2011). The current density in our protocol (0.04 mA/cm^2^) is very similar to that used previously (Reis et al., 2009; Schambra et al., 2011; Zimerman et al., 2013), and our stimulation duration of 20 min falls within the commonly used range (Reis et al., 2009; Schambra et al., 2011; Zimerman et al., 2013) (see Table 1).

Regarding the tDCS electrode montage, most previous tDCS studies have utilized the contralateral tDCS montage, i.e., with the reference electrode placed on the contralateral supraorbital area. Here we used an extracephalic ipsilateral reference electrode montage (from now on called the ipsilateral montage) that has been shown to enhance focality (Wagner et al., 2007; Datta et al., 2009; Sadleir et al., 2010). The ipsilateral montage has been shown to reliably change the cortical excitability of M1 as measured by TMS (Cogiamanian et al., 2007; Moliadze et al., 2010). However, empirical data suggest that it might be less beneficial for motor skill learning than the contralateral montage (Schambra et al., 2011). This hypothesis was put forward because (i) the effect size of motor learning gains was smaller (Schambra et al., 2011) and (ii) the effect on corticomotor excitability was lower with the ipsilateral compared to the contralateral montage when an intensity of 1 mA was used (Moliadze et al., 2010). However, this line of reasoning has to be considered with care because effect size was compared across studies that investigated different cohorts of subjects and, to the best of our knowledge, no study has demonstrated that larger changes in corticomotor excitability induced by anodal-tDCS are predictive of larger effects on motor learning.

In summary, the lack of multisession tDCS studies makes it difficult to compare our present results to other anodal-tDCS studies that have also targeted M1 to enhance motor learning. Furthermore, different methodological protocols have been used in the tDCS literature. We stimulated the right hemisphere with 1 mA for 20 min while training by using an ipsilateral montage. In most cases, anodal-tDCS is applied in combination with motor practice, targets the left-hemisphere, stimulates at 1 mA intensity for 15–20 min (current density between 0.02 and 0.04 mA/cm^2^) and uses a contralateral montage (for more details, see Table 1).

Nonetheless, the positive effects of anodal-tDCS reported here are in healthy subjects that have an intact motor system. It is possible that when applied to participants with impaired motor systems, e.g., due to aging or neurological disease, this effect might be bigger (Zimerman et al., 2013), providing an optimistic view on the implementation of this technique during rehabilitation.

## Conclusion

Our results support the hypothesis that the effect of anodal-tDCS over M1 on motor learning is task-specific. We argue that the nature of the task determines which brain areas contribute to the different processes mediating learning and memory formation. Consequently, anodal-tDCS will preferentially influence processes located in the area underneath the electrode, while effects on remote areas are probably too weak to become functionally relevant.

## Conflict of Interest Statement

The authors declare that the research was conducted in the absence of any commercial or financial relationships that could be construed as a potential conflict of interest.
